# The rediscovery of the rare Vietnamese endemic *Eriophorum scabriculme* redefines generic limits in the Scirpo-Caricoid Clade (Cyperaceae)

**DOI:** 10.7717/peerj.7538

**Published:** 2019-09-25

**Authors:** Julian R. Starr, Étienne Léveillé-Bourret, Vũ Anh Tài, Nguyê˜n Thị Kim Thanh, Bruce A. Ford

**Affiliations:** 1 Department of Biology, University of Ottawa, Ottawa, Ontario, Canada; 2 Institute of Systematic and Evolutionary Botany, University of Zürich, Zürich, Switzerland; 3 Institute of Geography, Vietnam Academy of Science and Technology, Hanoi, Vietnam; 4 Department of Botany, VNU University of Science, Hanoi, Vietnam; 5 Department of Biological Sciences, University of Manitoba, Winnipeg, Manitoba, Canada

**Keywords:** Vietnam, *Trichophorum*, *Eriophorum*, Conservation, Embryology, Phylogeny, Scirpus, Morphology, Cyperaceae, cpDNA

## Abstract

For those familiar with boreal bogs and wet tundra, species of *Eriophorum* (“the cotton grasses”) will undoubtedly represent some of the most striking and memorable taxa they have encountered. This small genus of 20 Holarctic sedge species (Cyperaceae) is remarkable because its inflorescences produce large, brilliantly white to rusty-red cottony masses when its flowers develop a perianth of highly elongated bristles after anthesis. In this study, we document the rediscovery of *Eriophorum scabriculme*, a narrow Vietnamese endemic known from only two collections made approximately 7 km apart near Sa Pa in Lào Cai Province over 75 years ago. Using plastid DNA sequences (*matK*, *ndhF*), embryology, and morphology, we test whether *E. scabriculme* is aligned within the Scirpo-Caricoid Clade (genus *Khaosokia* and tribes Cariceae, Dulichieae, Scirpeae, and Sumatroscirpeae) or the Ficinia Clade (Cypereae), and we determine whether its unique character combinations (≥10 elongated bristles, reduced sheathing basal leaves, 1–4 spikelets) could be evidence for a new genus or simply mark it as an unusual species within currently recognised genera. In addition, we document the discovery of seven new populations, and we extend its range westward to Lai Châu Province and southward in Lào Cai Province by more than 47 km. Our results demonstrate that *Eriophorum scabriculme* is best treated in the genus *Trichophorum*, thus re-circumscribing both genera and their limits with *Scirpus* s.str. In addition, we emend the description of *Trichophorum scabriculme* (Beetle) J.R.Starr, Lév.-Bourret & B.A. Ford, provide the first pictures and accurate illustration of the species, and assess its conservation status in Vietnam (VU, Vulnerable). Our study corroborates the fact that in such a diverse and taxonomically difficult family like the sedges, conspicuous characters like highly elongated bristles may be useful for dividing diversity, but they are no guarantee that the groups they mark are natural.

## Introduction

For those familiar with boreal bogs and wet tundra, species of *Eriophorum* L. (“the cotton grasses”) will undoubtedly represent some of the most striking and memorable taxa they have encountered. This small group of 20 Holarctic sedge species (Cyperaceae Juss.) is remarkable because its compound to unispicate infructescences form large, silky white to rusty-red cottony masses due to perianth bristles that elongate after anthesis. These bristles remain attached to the fruits and are probably involved in wind dispersal (Kern, 1974; Fridriksson, 1982; Goetghebeur, 1998; J Starr, pers. obs., 2015) and possibly heat retention (Simonis et al., 2014; Small & Cayouette, 2016). They also make *Eriophorum* one of the easiest of sedge genera to recognise for amateurs and professionals alike. Nevertheless, the circumscription of the genus remains unclear. Conspicuous characters may be useful for dividing sedge diversity, but there is no guarantee that the groups they mark are natural (Starr, Harris & Simpson, 2004; Larridon et al., 2013; Starr, Janzen & Ford, 2015).

The circumscription of *Eriophorum* is intimately linked to the circumscription of the genus *Scirpus* L., its closest ally and a taxon with a difficult taxonomic history. In the broad sense, *Scirpus* consisted of a heterogeneous group of over 250 species and more than 50 modern genera (Koyama, 1958; Schuyler, 1971; Dhooge, 2005), whose defining features are now believed to be plesiomorphies (spirally arranged scales, bisexual flowers with perianth parts absent or reduced to bristles and scales, and terete spikelets) (Goetghebeur, 1986; Muasya et al., 2009; Léveillé-Bourret & Starr, 2019). *Eriophorum* shared these characteristics with *Scirpus* s.l., but with exception of Koyama (1958), it was not placed in the synonymy of *Scirpus* largely because any sedge with long bristles and scirpoid features could be conveniently positioned within *Eriophorum*.

The limits of *Eriophorum* and *Scirpus* became clearer when evidence from embryo types (Van der Veken, 1965), fruit epidermal silica bodies (Schuyler, 1971) and inflorescence structure (Bruhl, 1995; Goetghebeur, 1986; Goetghebeur, 1998) began to suggest that *Scirpus* s.l. should be divided into multiple, often distantly related genera. The first molecular phylogenies for Cyperaceae (Muasya et al., 1998; Muasya et al., 2000) confirmed this, and the definition of *Scirpus* was eventually restricted to a Holarctic genus consisting of ca. 50 species that shared a Fimbristylis-type embryo (i.e., when known), noded culms, small spikelets in open anthelodia, and generally six perianth parts in the form of bristles when present (Dhooge, 2005; Muasya et al., 2009; Léveillé-Bourret & Starr, 2019). Nonetheless, the limits of *Eriophorum* remained unsatisfactory because only one character, bristle number, seemingly provided a reliable means for separating it from *Scirpus* (0 or ≤6 = *Scirpus*; ≥10 = *Eriophorum*; Goetghebeur, 1998). As family relationships became ever clearer, the most problematic taxa blurring their limits were slowly eliminated such that a focus on an even narrower range of taxa was possible. Studies demonstrated that morphologically confusing species of *Scirpus* or *Eriophorum* were separate generic lineages related either to elements in the distantly related Ficinia Clade of tribe Cypereae (Muasya et al., 2012; Yano et al., 2012; García-Madrid et al., 2015), or closely allied to *Scirpus* and *Eriophorum* within the Scirpo-Caricoid Clade or “SCC” (i.e., the Cariceae-Dulichieae-Scirpeae Clade, renamed for recent changes in tribal classification; Léveillé-Bourret, Starr & Ford, 2018; Léveillé-Bourret & Starr, 2019), a cosmopolitan group of more than 2,250 species and eight major lineages (Dulichieae, *Khaosokia*, *Calliscirpus*, Cariceae, Sumatroscirpeae, Trichophorum Clade, Zameioscirpus Clade, and Scirpus+Eriophorum Clade; Dhooge, Goetghebeur & Muasya, 2003; Gilmour, Starr & Naczi, 2013; Léveillé-Bourret et al., 2015; Semmouri et al., 2019).

These close relatives in the SCC represent the biggest problem for circumscription because they often possess seemingly intermediate characteristics. Over the past 30 years, five of these close allies have been recognised as separate genera. *Calliscirpus* C.N. Gilmour, J.R. Starr & Naczi, which blurred the limits of *Scirpus* and *Eriophorum* because it possesses lightly scabrous bristles (normally smooth in *Eriophorum*) intermediate in length and variable in number (6, rarely up to 12), is now known to represent a relatively distant lineage in the SCC (Gilmour, Starr & Naczi, 2013; Léveillé-Bourret et al., 2014; Léveillé-Bourret & Starr, 2019). In contrast, the recent *Scirpus* segregates *Zameioscirpus* Dhooge & Goetgh. and *Rhodoscirpus* Lév.-Bourret, Donadío & J.R.Starr are part of a molecularly and morphologically well-supported clade that includes *Amphiscirpus* and *Phylloscirpus* as sister to *Scirpus* and *Eriophorum*. Although *Zameioscirpus*, *Amphiscirpus*, and *Phylloscirpus* are morphologically distinctive (generally small and reduced, basal leaves, capitate to unispicate inflorescences), the gross morphology of *Rhodoscirpus* (e.g., large size, cauline leaves, open anthelae) resembles *Scirpus* s.str. to such a degree that the modern limits of *Scirpus* may still need to be redefined (Léveillé-Bourret et al., 2015; Léveillé-Bourret & Starr, 2019). The remaining two genera, *Cypringlea* M.T. Strong and *Oreobolopsis* T.Koyama & Guagl., appear to be aligned with *Trichophorum* Pers. (Strong, 2003; Dhooge, Goetghebeur & Muasya, 2003; Léveillé-Bourret et al., 2014; Semmouri et al., 2019), another genus that has been closely linked to the circumscription of *Scirpus* and *Eriophorum*.

Although *Trichophorum* has features that can separate it from *Scirpus* and *Eriophorum*, such as a reduced habit and sheathed basal leaves with poorly developed blades, its species share many important characteristics with *Scirpus* and *Eriophorum*. For example, *Trichophorum* species may or may not possess six or fewer short, serrrulate bristles as in *Scirpus*, and at least one species, *T. schansiense* Handel-Mazzetti, is reported to have more than six bristles (7–9) like an *Eriophorum* (Liang & Tucker, 2010b). Another species, *T. alpinum* (L.) Pers., has six bristles like a *Scirpus*, but they are long, silky and flattened like an *Eriophorum*, which explains why it has long been treated as a *Scirpus* and an *Eriophorum* (Schuyler, 1971). Moreover, inflorescences in the genus typically consist of a single terminal spikelet similar to *Eriophorum* p.p. (e.g., *E. vaginatum* L.), but a few short-bristled Southeast Asian species recently transferred from *Scirpus* can have as many as four to six (i.e., *T. subcapitatum* (Thwaites & Hooker) D. A. Simpson and *T. mattfeldianum* (Kükenthal) S. Yun Liang; Simpson, 1998; Fu, 2009).

Even their ecology suggests a close affinity. All three genera are common in boreal bogs, although only *Eriophorum* and *Trichophorum* grow on arctic and alpine tundra, and unlike either *Scirpus* or *Eriophorum*, a few *Trichophorum* are found on rock faces and ledges (Lee & Oh, 2006; Jung & Choi, 2010; J Starr, pers. obs., 2017), an unusual habitat for sedges. Nonetheless, despite the many interesting similarities noted above, *Trichophorum* differs markedly in its embryology, which is often a diagnostic character for determining generic or even tribal affinities in sedges (Goetghebeur, 1986; Goetghebeur, 1998; Semmouri et al., 2019). Whereas *Trichophorum* shares a Carex-type embryo with its close allies *Cypringlea* and *Oreobolopsis* (Van der Veken, 1965; Strong, 2003; Dhooge, 2005), *Scirpus* and *Eriophorum* possess a Fimbristylis-type embryo (Van der Veken, 1965; Goetghebeur, 1986), suggesting that ultimately they are more closely related to each other than to *Trichophorum*.

Molecular data has since confirmed there is a close relationship between *Eriophorum* p.p. and *Scirpus* s.str. (Gilmour, Starr & Naczi, 2013; Léveillé-Bourret et al., 2014). Of the four *Eriophorum* subgenera currently recognised (*Eriophorum*, *Eriophoropsis* (Palla) Raymond, *Phyllanthela* (Anderss.) Egor., *Erioscirpus* (Palla) Raymond; Novoselova, 1994a; Novoselova, 1994b), species from the temperate subgenera *Eriophorum*, *Eriophoropsis*, and *Phyllanthela* form a strongly supported clade nested in *Scirpus*. Whether these subgenera are best treated in *Scirpus* remains to be seen (Léveillé-Bourret et al., 2015; Léveillé-Bourret & Starr, 2019), but their monophyly is strongly corroborated by other data such as morphology (a simple anthela or a single spikelet, >10 long, smooth, perianth bristles), embryology (Fimbristylis-type embryo), ecology (bogs, tundra) and bristle development (Mora-Osejo, 1987; Vrijdaghs et al., 2005).

In contrast, the members of *Eriophorum* subgenus *Erioscirpus* (Palla) Raymond, a small, tropical to subtropical Southeast Asian group of four species (*E. scabriculme* (Beetle) Raymond, *E. comosum* (Wall.) Nees in R.Wight, *E. microstachyum* Boeckeler, *E. transiens* Raymond), differ markedly from all other *Eriophorum* in morphology (e.g., bristles barbed; Goetghebeur, 1998; J Starr, pers. obs., 2013), embryology (Van der Veken, 1965; Goetghebeur, 1986; Bruhl, 1995) and ecology, as all species are common to rocky slopes and walls (Raymond, 1957; Raymond, 1959a; Noltie, 1994; Liang, Tucker-Drob & Simpson, 2010; J Starr, pers. obs., 2015, 2017). In agreement with their atypical features for *Eriophorum*, molecular data has recently demonstrated that *E. comosum* and *E. microstachyum* are closely related to *Scirpus* segregates in the Ficinia Clade of tribe Cypereae and are thus best treated as a separate genus, *Erioscirpus* Palla (Yano et al., 2012). *Eriophorum transiens* is only known from its type (*J. Esquirol* 4367, P!), but its small spikelets arranged in dense glomerules, its long wispy bracts, and basally fused barbed bristles (Raymond, 1959a; Noltie, 1994; J Starr, pers. obs., 2013) suggest an affinity to species in the Ficinia Clade like several of the *Scirpus* segregates noted above. Consequently, only *Eriophorum scabriculme* still blurs the limits between *Eriophorum* and *Scirpus*.

*Eriophorum scabriculme* is a narrow Vietnamese endemic that is known from only two collections made approximately seven km apart near Sa Pa in Lào Cai Province over 75 years ago (*Pételot* 6128 & 8636, GH!, MT!, P!, L!). Unlike the other members of *Eriophorum* subg. *Erioscirpus*, *E. scabriculme* shows a clear morphological affinity to species in the SCC. Like *Eriophorum*, it possesses numerous (≥10) long, silky bristles, but similar to *Trichophorum*, it is a small plant with sheathing basal leaves and reduced blades that has a limited number of spikelets (1–4) in its inflorescence. Moreover, like a few *Trichophorum* species, it lives on rocky vertical habitats like slopes and cliffs.

In this study, we document the re-discovery of *Eriophorum scabriculme* during two field seasons in Northern Vietnam. Using DNA sequences, embryology, and morphology we test whether *E. scabriculme* is best treated as an *Eriophorum* or in allied genera, whether it should be aligned with *Erioscirpus* species in the Ficinia Clade, or whether its unique character combinations could be evidence for a new generic lineage in Cyperaceae. The results of our analyses clearly demonstrate that *E. scabriculme* is not related to either *Erioscirpus* species or to other *Eriophorum*, but represents an unusual species in the genus *Trichophorum*. We thus transfer the species to the genus *Trichophorum* as *T. scabriculme* (Beetle) J.R. Starr, Lév.-Bourret & B.A. Ford, clarifying the limits of both *Trichophorum* and *Eriophorum*. In addition, we document the discovery of seven new populations, extending its range to over 47 km, including two populations from Lai Châu, the province adjacent to Lào Cai. We emend the description of *T. scabriculme*, provide an illustration of the species, assess its conservation status, and provide a key to the *Trichophorum* in Vietnam and southern China.

## Materials & Methods

### Collection and exportation permits

All Vietnamese samples used in this study were collected and exported under permit numbers 222/TCLN-BTTN (2012) and 184/TCLN-BTTN (2015), which were issued by the Vietnamese Forest Protection Department with the support of the faculty and staff at VNU University of Science, Hanoi. Vouchers for all taxa used in this study are available in publicly accessible herbaria (Table S1).

### Taxonomic sampling, molecular markers, and outgroup selection

To determine the generic position of *Eriophorum scabriculme* (hereafter referred to as *Trichophorum scabriculme*), taxa were selected to fully represent the morphological and geographical diversity of all eight major lineages discovered in previous molecular analyses focused on the SCC (Gilmour, Starr & Naczi, 2013; Léveillé-Bourret et al., 2015; Léveillé-Bourret et al., 2014; Léveillé-Bourret, Starr & Ford, 2018) (Table S1). To test whether *Trichophorum scabriculme* could be related to *Erioscirpus* as suggested by many previous authors (Raymond, 1957; Koyama, 1958; Novoselova, 1994a), *Erioscirpus comosus* was included in all analyses. Portions of the rapidly evolving plastid genes *matK* and *ndhF* were used in all analyses as these markers have been shown to be highly useful for determining generic placement within the SCC (Gilmour, Starr & Naczi, 2013; Léveillé-Bourret et al., 2015; Léveillé-Bourret et al., 2014). Outgroups were selected using the results of previous family-wide analyses of Cyperaceae (Muasya et al., 1998; Muasya et al., 2009).

### DNA Extraction, PCR amplification, sequencing and alignment

Genomic DNA was extracted from herbarium or field-collected (silica-dried) leaf tissue following the silica-column based protocol of Alexander et al. (2007) as modified by Starr, Naczi & Chouinard (2009). PCR and sequencing protocols for *matK* and *ndhF* follow Gilmour, Starr & Naczi (2013) and Léveillé-Bourret et al. (2014).

Sequence chromatograms were assembled and corrected in Geneious 8.1.9. Alignments were made using MAFFT version 7.017b (Katoh & Standley, 2013), concatenated by species, and then adjusted by hand using parsimony as an objective criterion (Starr, Harris & Simpson, 2004).

### Phylogenetic analyses

Both parsimony and maximum likelihood (ML) analyses were conducted on the combined *matK* + *ndhF* matrix. For parsimony analyses, heuristic searches were conducted in PAUP* version 4.0b10 (Swofford, 2003) using a random addition sequence of taxa for 2,000 replicates with the MULTREES option on and no more than 10 trees saved per replicate. Relationships were evaluated from a strict consensus tree produced in PAUP*. Branch support was evaluated from 10,000 heuristic bootstrap replicates with the MULTREES option turned off (DeBry & Olmstead, 2000), and the level of BS support was subjectively described as follows: strong 95–100% BS; very good 85–94% BS; good 75–84% BS; moderate 65–74% BS; weak 55–64% BS; and very weak <55% BS (Starr, Harris & Simpson, 2004).

Concatenated ML analyses were performed with RAxML version 8.2.12 (Stamatakis, 2014). The partitioning scheme was selected among all codon and locus subsets with PartitionFinder version 2.1.1 (Lanfear et al., 2012) constraining the model for each partition to be either GTR or GTR+G, and using RAxML for partitioning scheme comparisons. RAxML searches were made with 200 randomized maximum parsimony starting trees and the old and slower, but more accurate, rapid hill—climbing algorithm (option -f o). Branch support was assessed with 2,000 standard bootstrap replicates (Felsenstein, 1985).

The *matK* + *ndhF* data matrix used in analyses is provided as supplemental information with this article (Data S1). DNA sequences were submitted to GenBank (Table S1).

### Morphological studies, botanical illustration and taxonomic key

The protologue for *Trichophorum scabriculme* (as *Eriophorum scabriculme*; Beetle, 1946) was based on an immature specimen, which explains why Raymond (1957) emended the description when he received more complete specimens years later. However, discrepancies between Raymond’s emended description and observations made by us in the field (e.g., bristle and anther number) suggested that a more thorough description of the species using all known material was necessary. The holotype and the specimens used by Raymond (1957) were included in the preparation of the description. In addition, a detailed illustration of *T. scabriculme* was made as one was not included with the protologue or in Raymond (1957). The only illustration for *Trichophorum scabriculme* (Phạm, 1993) does not appear to have been made from a live plant or specimen.

A key to the genus *Trichophorum* in Vietnam and southern China was developed from the literature and specimen data. *Trichophorum subcapitatum* is part of a difficult and variable East and Southeast Asian group that has been variously treated by authors, with distinguishing characters often subtle and intergrading (Beetle, 1946; Kern, 1956; Kern, 1974; Liang & Tucker, 2010b). It includes *Trichophorum mattfeldianum*, which is segregated based on the presence of (obtusely) trigonous culms, but most specimens of the group have at least slightly rounded-trigonous culms near the base, and this characteristic does not appear to be correlated with other morphological differences, despite claims to the contrary. A revision of the morphological variation of the complex, which includes *Scirpus clarkei* Stapf., *S. morrisonensis* Hayata, *S. pulogensis* Merr., *S. pakapakensis* Stapf. and *S. subcapitatus* subsp. *celebicus* J.Kern, is therefore needed before a taxonomic system can be proposed. We thus refer all these taxa to the “*Trichophorum subcapitatum* agg.” in our key.

### Embryology

Embryo morphology is well characterised in the SCC (Van der Veken, 1965; Goetghebeur, 1986; Dhooge, 2005; Gilmour, Starr & Naczi, 2013; Semmouri et al., 2019), and it is an excellent tool for testing hypotheses of generic and tribal placement when used in conjunction with other data. Embryos from *Trichophorum scabriculme* were prepared and visualised following Gilmour, Starr & Naczi (2013) and the embryo type was determined following the type designations described in Goetghebeur (1986) and Dhooge (2005).

### Range and habitat descriptions

All known populations and collections of *Trichophorum scabriculme* were mapped using GPS coordinates or specimens that could be georeferenced to <one km area of uncertainty (a few exceptions are noted below under Conservation Status). Collections were only considered to represent separate populations when contiguous collections were more than one km part. Multiple collections made near Ô Quy Hồ Pass and Tiên Sa Waterfall were thus considered to be just two populations. Habitat descriptions for *T. scabriculme* were characterised from specimen labels and field experience in Vietnam.

### Conservation status

The conservation status of *Trichophorum scabriculme* was assessed according to the criteria outlined in IUCN Red List Categories and Criteria: Version 3.1 (IUCN, 2012). GeoCAT (Bachman et al., 2011) was used to determine the extent of occurrence for *T. scabriculme* based on specimens that represented unique localities with GPS coordinates or specimens that could be georeferenced to a <one km area of uncertainty with three exceptions. These included a specimen from Lai Châu Province (N.T. Hiep, P.H. Hoang & L. Averyanov 2846, MO) whose locality was roughly estimated from label data because it represented one of only two populations known from this province, and two observations of individuals that were too immature or too mature to warrant collecting, but for which georeferenced pictures (GPS) were taken. Full locality data for the two observations without collections is given under the “Notes” section of the Taxonomic Treatment below. This data is also available in the GeoCAT file of all documented localities that was submitted as Data S2. The number of unique populations known to occur within or outside protected areas was determined using the World Database on Protected Areas (IUCN & UNEP, 2014) as a layer in GeoCAT.

## Results

### Phylogenetic analyses

The alignment of *matK* + *ndhF* sequences for 72 taxa produced a data matrix of 2,534 characters of which 1,671 were constant and 513 were parsimony informative. Heuristic parsimony searches recovered 3,346 most parsimonious trees of 1,721 steps (CI = 0.63; RI = 0.76). The best scoring scheme in PartitionFinder (BIC = 28,360.2879946) included two GTR+Gamma partitions: (1) codon positions 1 and 2 of *matK* and *ndhF*, and (2) codon position 3 of *matK* and *ndhF*. The best-scoring ML tree had a log-likelihood of −13,582.242449.

**Figure 1:**
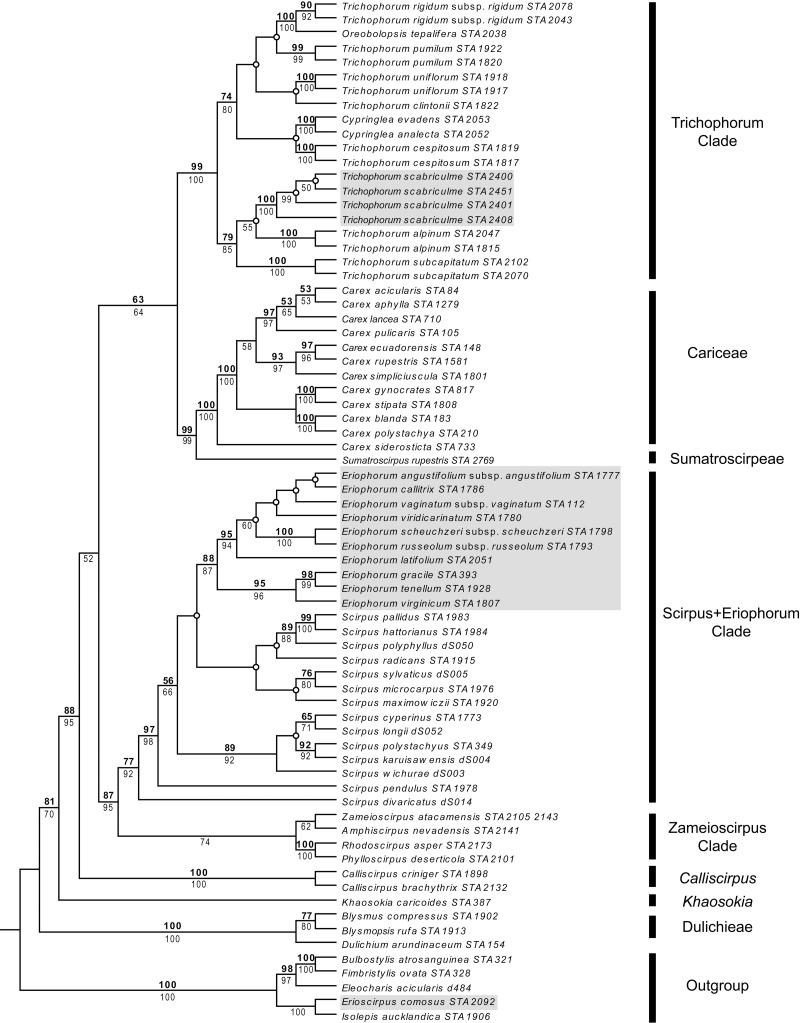
Maximum likelihood (ML) tree resulting from the combined analysis of *matK* and *ndhF* plastid sequences. Numbers above branches are parsimony bootstrap values and numbers below branches are ML bootstrap values. Open circles represent branches that collapsed in the parsimony strict consensus. The names of major Scirpo-Caricoid Clades (see text) are given to the right of species names. Numbers after species epithets correspond to specific vouchers in Table S1. Samples of *Trichophorum scabriculme* are shaded in grey to illustrate their position in the Trichophorum Clade as are species of *Eriophorum* s.str. and *Erioscirpus comosus* to highlight their clear separation from *T. scabriculme.*

Parsimony and ML analyses were fully compatible with the ML tree being more resolved than the strict consensus of parsimony analyses. For example, both analyses placed *Trichophorum alpinum*, the *T. subcapitatum* agg. and *T. scabriculme* in the same clade with good support, but whereas ML analyses had *T. alpinum* as sister to *T. scabriculme* (ML-BS 55%), this node was lacking in the strict consensus of parsimony analyses. Since these differences did not affect the interpretation of results, the more resolved ML tree is presented with parsimony and ML bootstrap values given above and below branches (Fig. 1). The subjective description of clade support was only based on the values for parsimony as support values are similar for each method of analysis.

The tree in Fig. 1 shows the eight major lineages identified in the analysis of Léveillé-Bourret, Starr & Ford (2018) with similar levels of support; namely, Dulichieae (*Blysmus*, *Dulichium*), *Khaosokia*, *Calliscirpus*, a Zameioscirpus Clade (*Phylloscirpus*, *Rhodoscirpus*, *Amphiscirpus*, *Zameioscirpus*), a *Scirpus* + *Eriophorum* Clade, Sumatroscirpeae, Cariceae and a Trichophorum Clade (*Cypringlea*, *Trichophorum*, *Oreobolopsis*). *Trichophorum scabriculme* is positioned within a strongly supported Trichophorum Clade, and within a subclade with good support consisting of the Southeast Asian *T. subcaptitatum* agg. and the circumboreal *T. alpinum*, which is the type for the genus (hereafter the “*Trichophorum scabriculme* Clade”) (Salmenkallio & Kukkonen, 1989). This clade is sister to a moderately supported clade of all other included *Trichophorum* species (circumboreal, South American, Asian), as well as species of *Cypringlea* (Mexican) and *Oreobolopsis* (South America), although relationships are not well supported or resolved. All sequenced individuals of *Trichophorum scabriculme* were placed in a single strongly supported clade and displayed no sequence variation in the markers chosen. Topologies clearly demonstrate that *T. scabriculme* is not a member of either the genus *Eriophorum* or *Erioscirpus*.

### Embryology

**Figure 2:**
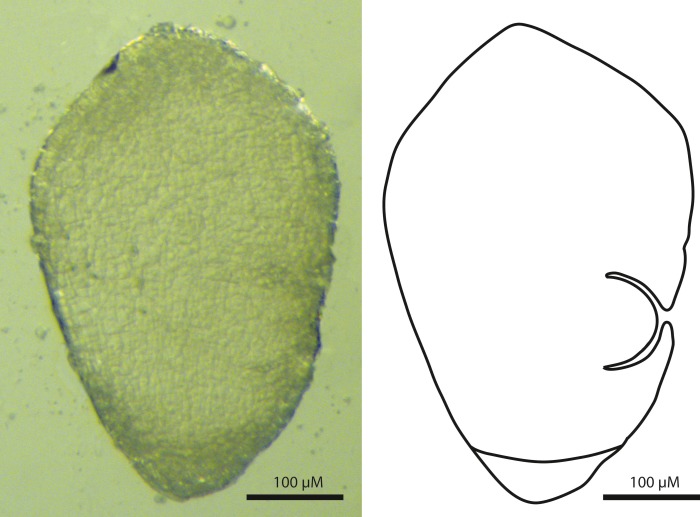
Sagittal view of the Carex-type embryo of *Trichophorum scabriculme* (*B. Ford 1225, J. Starr & J. Regalado*, WIN). Scale bar = 100 µm.

The embryos of *T. scabriculme* were turbinate (top-shaped) in outline, and possessed a basally positioned root cap and a laterally positioned first leaf (Fig. 2). This clearly identifies them as a Carex-type embryo (Van der Veken, 1965; Goetghebeur, 1986; Dhooge, 2005; Semmouri et al., 2019).

### Morphology

**Figure 3:**
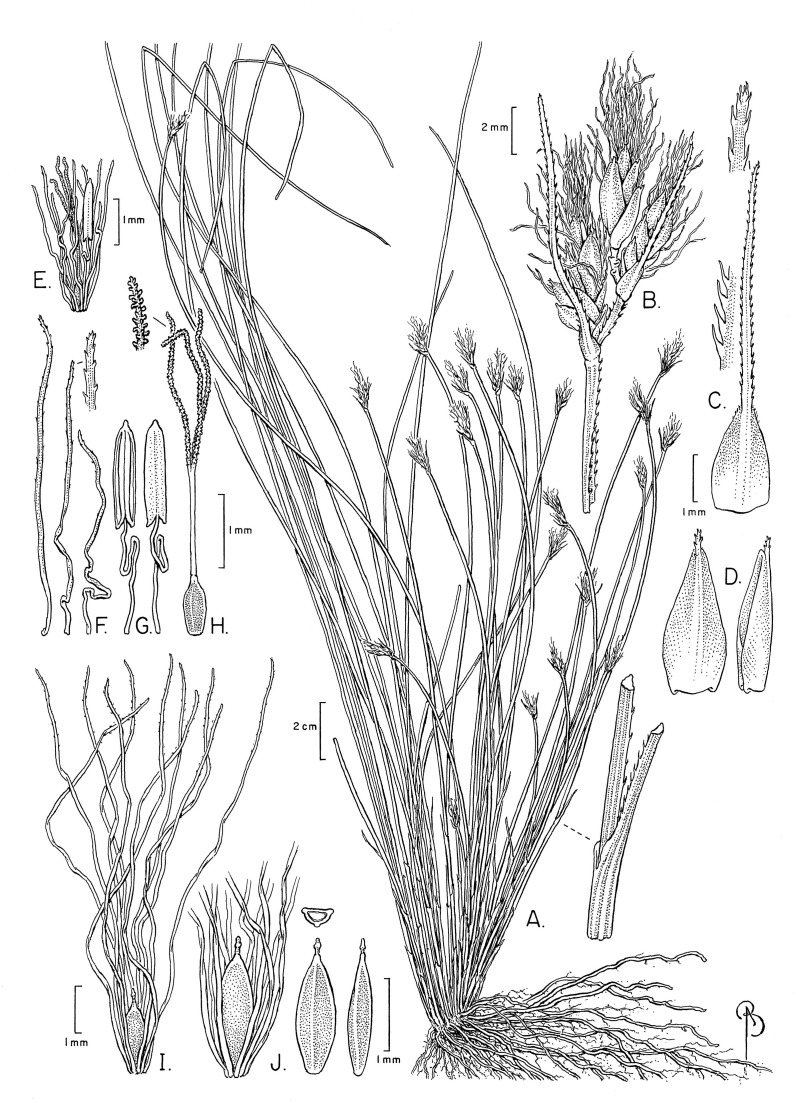
Illustration of *Trichophorum scabriculme*. (A) Habit. (B) Inflorescence with three spikelets. (C) Bract with antrorsely scabrous awn. (D) Glume (proximal) with awn, abaxial and lateral views. (E) Flower with developing bristles (note single stamen). (F) Bristles, showing minute distally antrorse barbs. (G) Stamen, adaxial and abaxial views. (H) Gynoecium (note style branches with abundant large papillae as long as wide). (I) Nutlet (mature) with full length bristles. (J) Nutlet (mature), close up with abbreviated bristles. Nutlet (mature), in cross-section and with abaxial and lateral views. Drawn from Ford 1227A & al. (WIN). Illustration by Bobbi Angell.

The specimens examined differed significantly in culm shape, and in the length and number of bristles as compared to the description of these characters in Beetle (1946) and Raymond (1957), and in the illustration of the species provided by Phạm (1993). The number of anthers (1) has not been reported before. These differences are discussed below under the section “Notable morphological differences with previous descriptions and illustrations”. An illustration (Fig. 3) and emended description of *T. scabriculme* is provided in the Taxonomic Treatment.

### Species distributions and habitat descriptions

**Figure 4:**
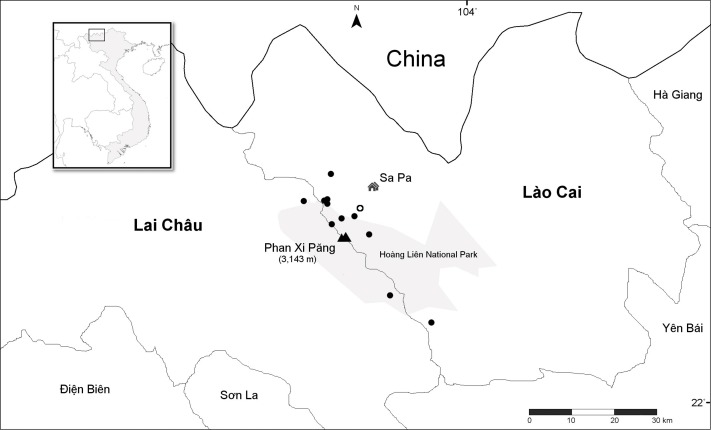
Distribution of *Trichophorum scabriculme* in the two northern Vietnamese provinces of Lai Châu and Lào Cai with China to the north. Closed circles represent collection sites with the open circle representing the type locality, “La Cascade”, which is known today as Tiên Sa Waterfall (see text). Hoàng Liên National Park is in grey with mount Phan Xi Păng and the town of Sa Pa indicated on the map. Inset shows the area covered relative to the whole of Indochina. Scale bar is for 30 Km. Map redrawn from SimpleMappr.

**Figure 5:**
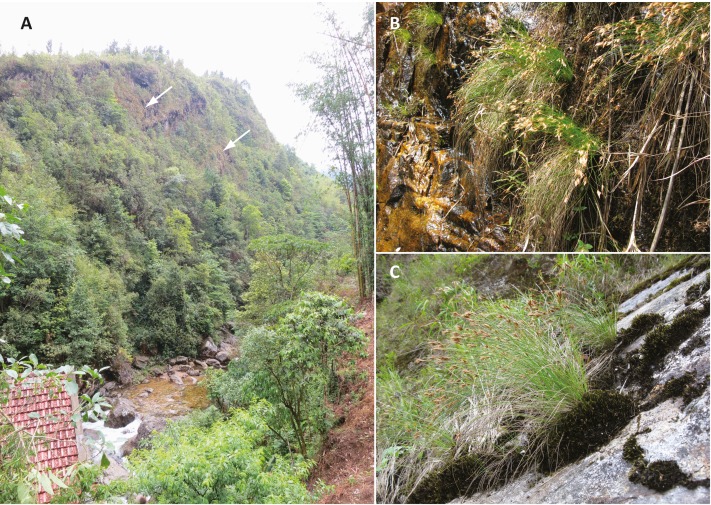
Habitat of *Trichophorum scabriculme*. (A) The largest population observed in Vietnam on the right bank of the suô´i Vàng (Gold Stream). The arrows indicate plants on cliff face. The picture is taken from the type locality for *Trichophorum scabriculme* (“La Cascade”) with the French power station constructed in 1925 visible in the lower left corner. (B) Plants growing in fissures on edge of waterfall. (C) Plants growing in mossy substrate. Photo credit: Julian Starr.

Nine unique populations were documented from 16 collections in two Vietnamese provinces and four Districts (Lào Cai Province, Sa Pa and Văn Bàn Districts; Lai Châu Province, Tam Ðường and Tân Uyên Districts) (Fig. 4). All populations are known from the Hoàng Liên Mountains (612 m to 2,878 m) in full sun on vertical rock walls and rocky road cuts, along steep cascading streams and around waterfalls (Fig. 5). Plants typically grow in a moist, mossy substrate.

### Conservation status and species distribution

**Figure 6:**
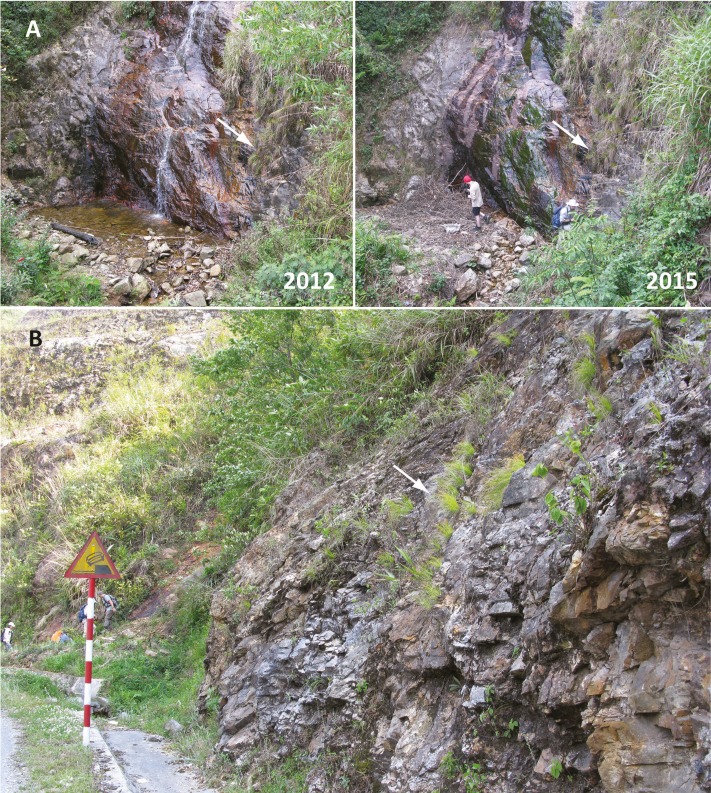
Anthropogenic threats to known populations of *Trichophorum scabriculme*. (A) Hydrological change (2012 versus 2015). (B) Threat of widening well-traveled roads. Photo credit: Julian Starr.

Five of nine total populations are within protected areas (Hoang Lien National Park), but all of these populations are heavily influenced by human activity (Fig. 6). The extent of occurrence of the species is 252.8 km^2^ over a north to south-south west axis of 47 km. A GeoCAT file containing all the specimens and georeferenced photos used to assess the extent of occurrence and conservation status of *Trichophorum scabriculme* are provided in Data S2. This file will be available on the first author’s website and it will be updated when further documented specimens are discovered.

## Discussion

### The limits of *Eriophorum* and *Trichophorum* are clarified by the transfer of *Eriophorum scabriculme* to *Trichophorum*

Despite its status as one of the oldest (Linnaeus, 1753) and most recognisable genera in Cyperaceae, confusion over how to circumscribe the genus *Eriophorum* has persisted to this day. This confusion is intimately related to the circumscription of *Scirpus* s.l., a heterogeneous taxon consisting of over 50 modern genera and more than 250 species (Koyama, 1958; Schuyler, 1971; Dhooge, 2005). *Eriophorum* shared the defining characteristics of *Scirpus* s.l., but authors consistently kept *Eriophorum* separate from *Scirpus*, except Koyama (1958), because any sedge with scirpoid features and long bristles could be conveniently placed within it, and most *Eriophorum* species were well known, north-temperate taxa with a clear morphological relationship.

The modern circumscription of *Scirpus*, which is based on multiple lines of evidence from embryo types (Van der Veken, 1965), fruit epidermal silica bodies (Schuyler, 1971) inflorescence structure (Bruhl, 1995; Goetghebeur, 1986; Goetghebeur, 1998) and molecular phylogeny (Muasya et al., 2009; Muasya et al., 2000), has considerably narrowed the problem. Recent studies have consistently demonstrated that morphologically confusing *Scirpus* or *Eriophorum* species were typically separate generic lineages related to either species in the distantly related Ficinia Clade of Cypereae (Muasya et al., 2012; Yano et al., 2012; García-Madrid et al., 2015), or closely allied to *Scirpus* and *Eriophorum* within the SCC, a cosmopolitan group comprising eight major lineages (Dulichieae, *Khaosokia*, *Calliscirpus*, Sumatroscirpeae, Cariceae, Trichophorum Clade, Zameioscirpus Clade, and Scirpus+Eriophorum Clade; Dhooge, Goetghebeur & Muasya, 2003; Gilmour, Starr & Naczi, 2013; Léveillé-Bourret et al., 2015; Léveillé-Bourret, Starr & Ford, 2018; Semmouri et al., 2019). Several of these lineages were clearly separate from *Scirpus* and *Eriophorum* and their allies in tribe Scirpeae, such as tribes Cariceae, Dulichieae and Sumatroscirpeae, and the morphologically unusual genus *Khaosokia*, but the relationships of the remaining elements were not clear because the limits of *Scirpus* and *Eriophorum* remained ill-defined. Moreover, molecular phylogenies suggested that tribe Scirpeae was paraphyletic with respect to the large and morphologically cohesive tribe Cariceae, which contains the well-known genus *Carex* L. (∼2,150 species; e.g., Léveillé-Bourret et al., 2014; Léveillé-Bourret et al., 2018). The segregation of *Zameioscirpus*, *Amphiscirpus*, *Phylloscirpus* and *Rhodoscirpus* (all in South America) from *Scirpus* (Oteng-Yeboah, 1974; Dhooge, Goetghebeur & Muasya, 2003; Léveillé-Bourret et al., 2015), and the transfer of the transitional *Eriophorum crinigerum* Beetle (six, sometimes more, lightly serrulate bristles of intermediate length) to a new genus *Calliscirpus* (Gilmour, Starr & Naczi, 2013), were key steps in clarifying the limits between *Scirpus* and *Eriophorum* because the challenge could now be restricted to relatively large, north-temperate plants. Even so, bristle number still provided the only reliable means for distinguishing species of *Scirpus* from those in *Eriophorum* (0 or ≤ 6 = *Scirpus*; ≥ 10 = *Eriophorum*; Goetghebeur, 1998).

Past attempts to reliably separate *Scirpus* and *Eriophorum* have combined bristle number with bristle length, or have tried to correlate these characters with other vegetative and reproductive features. A prime example of how changing generic concepts have failed to draw a reliable line between *Scirpus* and *Eriophorum* is provided by *Scirpus maximowiczii* C.B. Clarke. In his protologue for the species, Clarke (1908) noted that except for its six lightly scabrous bristles just surpassing the nutlet, *S. maximowiczii* had more in common with species of *Eriophorum*. Subsequent authors agreed that when bristle length was assessed within the context of its habitat preferences (wet montane meadows), achene size, and vegetative morphology (e.g., large pendulous spikelets, scarious blackish glumes), *S. maximowiczii* was best placed in the genus *Eriophorum* (Beetle, 1946; Koyama, 1958; Oteng-Yeboah, 1974). Today, however, treatments universally place this species in *Scirpus* (e.g., Egorova, 2004; Liang & Tucker, 2010a; Hoshino, Masaki & Nishimoto, 2011) because the line between *Scirpus* and *Eriophorum* is again focused on bristle number. The failure to find generic characters that could reliably divide species into either *Scirpus* or *Eriophorum* can now be explained by fact that *Eriophorum* may be monophyletic, but it is deeply nested in *Scirpus* (Gilmour, Starr & Naczi, 2013; Léveillé-Bourret et al., 2014). Consequently, it would appear that the defining features for *Scirpus* s.str. are plesiomorphies (Léveillé-Bourret & Starr, 2019). Whether the distinctive genus *Eriophorum* should be maintained awaits further study, but it would require the naming of at least six to eight new genera to make *Scirpus* monophyletic (Léveillé-Bourret et al., 2015).

The strong focus on bristle number and length in the circumscription of *Eriophorum* and *Scirpus* is the only reasonable way to explain why past authors placed *Erioscirpus* species, *Trichophorum scabriculme* and *Eriophorum transiens* in *Eriophorum*. They may share long, numerous bristles, a common distribution, and a somewhat similar habitat (rock faces; though *Erioscirpus comosus* = dry, *Trichophorum scabriculme* = humid; J Starr, pers. obs., 2012, 2015), but they share very little else with each other or *Eriophorum* s.str. (Table 1). Even the character that seemingly unites them the best, their long, numerous bristles, appears to be no indication of relationship. We can confirm from direct observations of wind dispersing fruits of *Trichophorum scabriculme* (*B. Ford 15053* et al. WIN) and *Erioscirpus comosus* (*B. Ford 1269* et al. WIN) that their distinctive bristles are most likely a convergent adaptation to wind dispersal (Kern, 1974; Goetghebeur, 1998; Yano et al., 2012).

**Table 1:** Comparison of important morphological characters of genera that have been associated with *Trichophorum scabriculme*.

**Character**	*Trichophorum*	*T*. *scabriculme*	*Scirpus* s.str.	*Eriophorum* s.str.	*Erioscirpus*
Roots disintegrating into tough black wiry strands	never	never	never	never	yes
Basal sheaths colour	green–yellowish brown	green–yellowish brown	green–yellowish brown	green–yellowish brown	dark brown/marroon
Leaves	basal	basal	basal and cauline	basal and cauline (sometimes basal only)	basal
Leaf blade	much reduced, rarely well developed	much reduced	well developed	well developed	well developed
Ligule	Present	Present	Present	Present	Absent
Basal bract length compared to inflorescence (when present)	shorter–longer	mostly longer	about equal	about equal	much longer
Inflorescence branch habit (when present)	upright	upright	upright–drooping	drooping	upright
Number spikelets per inflorescence	1–6	1–3	50+	1–3(–30)	1–50+
Cauline leaves	0	0	many	(0–)many	0
First glume differentiation	mostly longer, mucronate or awned	longer, awned	undifferentiated	undifferentiated	undifferentiated
Sterile proximal glume(s)	1(–2)	1	0	0(–many)	0
Number perianth parts	0–6	10–14	0–6	≥10	>10
Perianth colour	whitish	reddish	whitish	white–red	grey
Perianth cross-section shape	±terete–flattened	flattened	±terete	flattened	±terete
Perianth barbs at apex	absent or “crown” of sharp teeth	“crown” of sharp teeth	absent or a few retrorse barbs forming a “harpoon”	“crown” of sharp teeth	2–3 divaricate teeth forming a “grappling-hook”
Perianth barbs below apex	absent or present, antrorse or patent, blunt or sharp	antrorse, sharp	Absent or present, antrorse or retrorse, sharp or blunt	absent	patent, blunt
Anther apiculum	small, oblong	small, oblong	small, oblong	small, oblong	long, acuminate
Embryo root cap position	basal	basal	lateral	lateral	lateral
Embryo germ pore orientation (in relation to first leaf)	perpendicular	perpendicular	perpendicular	perpendicular	parallel

In contrast, the position of *Trichophorum scabriculme* within the Trichophorum Clade and in a monophyletic group with *T. alpinum* and the *T. subcapitatum* agg. is entirely consistent with embryology, morphology, and biogeography. The *Carex*-type embryo of *T. scabriculme* is common to all species in the SCC except the Eriophorum + Scirpus and Zameioscirpus Clades, sister groups that share the morphologically similar Fimbristylis- and Schoenus-type embryos (Van der Veken, 1965; Strong, 2003; Dhooge, 2005; Gilmour, Starr & Naczi, 2013). Morphologically, the position of *Trichophorum scabriculme* within a clade that includes the *T. subcapitatum* agg. is consistent with the fact that these are the only taxa in *Trichophorum* to have more than one spikelet per inflorescence and they are among the few species in *Trichophorum* to grow on damp rock faces and ledges (Zhen, Liang & Tucker, 2010; J Starr, pers. obs., 2017). They also share antrorsely scabrous reddish bristles and they are confined to the mountainous regions of East and Southeast Asia with considerable overlap in their ranges (Liang & Tucker, 2010b). Even the position of the unispicate *T. alpinum* in a largely paucispicate clade and sister to *T. scabriculme* in ML analyses is coherent because *T. alpinum* and *T. scabriculme* are the only species in the genus to possess bristles greater than ten times the length of the nutlet. Most *Trichophorum* species have bristles that are rarely more than twice the fruit’s length and several lack bristles altogether (Liang & Tucker, 2010b).

With the addition of *T. scabriculme* to *Trichophorum*, the genus now consists of 13 species plus a fourteenth, *Trichophorum dioicum* (Lee & Oh) J.Jung & H.K.Choi, whose taxonomy and nomenclature needs to be resolved (see Lee & Oh, 2006; Lee & Oh, 2007; Jung & Choi, 2010). The genus *Trichophorum* can be separated from other Cyperaceae by its reduced, basal sheathing leaves with ligules, blades much reduced (rarely developed), spikelets solitary or in paucispicate racemes (rarely), flowers surrounded by a perianth of 6 (—14) bristle-like tepals (rarely absent), antrorsely barbed or smooth (rarely), deciduous with a fruit containing a Carex-type embryo, and with the proximal glume of spikelets sterile (fertile in *T. cespitosum*), differentiated, and frequently awned.

Species of *Trichophorum* can be separated into two distinct clades with good support, the *T. scabriculme* Clade described above, which contains the type *T. alpinum*, and its sister clade, a poorly resolved group of all other *Trichophorum* plus the genera *Cypringlea* and *Oreobolopsis*. Although these two major clades within the Trichophorum Clade have received no more than 73—79% BS support in previous analyses, they present the real possibility that *Cypringlea* and *Oreobolopsis* species may need to be transferred to *Trichophorum* or that new genera will need to be created to maintain generic monophyly. *Oreobolopsis* species fit well into *Trichophorum* as they are very similar to species with an inflorescence consisting of a single terminal spikelet, the only remarkable difference being the presence of six broad, membranous tepals in flowers instead of bristles. However, the value of this single perianth character to segregate *Oreobolopsis* from *Trichophorum* is questionable (Dhooge, 2005), especially when it appears that glume-like perianth parts are not entirely unknown in species like *T. subcapitatum* (see Léveillé-Bourret et al., 2014), a close relative of the generic type *T. alpinum*. *Cypringlea* also shares several characteristics with *Trichophorum* such as mostly basal leaves, antrorse to divergent bristle barbs and spikelets with sterile basal glumes, but it possesses well-developed leaves and simple to compound anthelae sometimes consisting of hundreds of spikelets (Strong, 2003; Reznicek & González-Elizondo, 2008; Léveillé-Bourret et al., 2014). Whether *Cypringlea* and *Oreobolopsis* are best treated in *Trichophorum* will require further analysis, but close relationships among multispicate, paucispicate, and unispicate species are common throughout most of the major SCC clades, suggesting that reduction and proliferation is a frequent mode of evolution among these lineages (Léveillé-Bourret et al., 2014).

With the removal of *Trichophorum scabriculme* and *Erioscirpus* species, the genus *Eriophorum* s.str. now consists of approximately 18 species (Novoselova, 1994b; Ball & Wujek, 2002; Léveillé-Bourret & Starr, 2019) with *E. transiens* an aberrant oddity characterised by spikelets arranged into an anthela of dense, globose heads with branches subtended by extremely long bracts and flowers with bristles fused at the base. This type of inflorescence suggests *E. transiens* is distantly related to *Eriophorum* and more likely to be allied to elements in the Ficinia Clade as analyses have shown for other recent *Scirpus* segregates (e.g., *Afroscirpoides* and *Dracoscirpoides*; Muasya et al., 2012; García-Madrid et al., 2015). The taxonomy and relationships of *E. transiens* are currently under study.

*Eriophorum* can now be separated from *Scirpus* and all other Cyperaceae by basal and cauline leaves with ligules, spikelets solitary and erect or in subcapitate to pendant anthelae of few (1—)2—10(—30), typically large spikelets with flowers surrounded by numerous (10–25) smooth, flattened long bristles that are white to rusty-red in colour, and with nutlets containing a Fimbristylis-type embryo. The genus is nested within *Scirpus* s.str. as currently understood, but further research is required to determine whether the limits of *Scirpus* are appropriately defined.

### Notable morphological differences with previous descriptions and illustrations

The protologue for *Trichophorum scabriculme* by *Beetle (1946)* was based on immature material collected early in the growing season (February). Consequently, no nutlets were present and the elongate bristles that characterise this species were not exsert from the glumes. This immaturity most likely explains why Beetle (1946) gives the number of bristles as six despite 10 to 14 being typical for the species because an accurate count could not be made and he believed the species was a *Scirpus*. Consequently, Raymond (1957) emended the description of *T. scabriculme* when he received better material from a second collection of the species by the holotype collector, Alfred Pételot (Raymond, 1955). This description also contains errors, some of which are probably related to the fact that the specimens used by Raymond were too mature. The differences between our observations and Raymond’s in bristle length (up to nine mm vs. 25 mm) and number (up to 24 versus 14) might be explained by the fact that the material at MT (A. Pételot 8635) had already lost many of its nutlets. As we observed efficient wind dispersal of *T. scabriculme* in the field, it is possible that those nutlets with the longest bristles may have already been dispersed by the wind before the plants were collected. Moreover, the unusual bristle numbers reported by Raymond could be due to extrapolation if he believed that many bristles had already been lost due to maturity.

There are other notable differences between our observations and those of Beetle (1946) and Raymond (1957). Whereas they recorded triangular stems for *Trichophorum scabriculme*, we observed crescentiform to fusiform culms in cross-section. In addition, neither author mentions anything about the stamens, which is understandable given the problems with specimen maturity noted above. *Trichophorum scabriculme* has only one stamen, a character apparently unique in *Trichophorum*, but not entirely uncommon in Cyperaceae (Goetghebeur, 1998).

The only illustration of *T. scabriculme*, which is provided in the Illustrated Flora of Vietnam (Phạm, 1993; reproduced in Khôi, 2002), appears to represent an image developed from the composite descriptions of Beetle (1946) and Raymond (1957). None of the plants on specimens available to Phạm in 1993 (Phạm, 1993) resemble the illustration, and characters such as bristle number (23) and length (four mm) appear to be compromises between the conflicting values given in Beetle (6 bristles, not exsert from glumes 3–4 mm long; Beetle, 1946) and Raymond (up to 24 bristles, 8–9 mm long, glumes 3–4 mm long; Raymond, 1957).

### Conservation status of *Trichophorum scabriculme*

Vulnerable (VU) category of IUCN (IUCN, 2012). Prior to this study, *Trichophorum scabriculme* was known from only two collections ca. seven km apart made near Sa Pa in Lào Cai Province during the French colonial period over 75 years ago. This is a famous locality for endemism in northern Vietnam that supports a diverse mixture of tropical, subtropical and temperate plants (Raymond, 1959b; Thin & Harder, 1996; Ford et al., 2015) owing to its mountainous topography which includes mount Phan Xi Păng (Fansipan; 3,143 m), the tallest peak in Indochina.

Here we report the rediscovery of *Trichophorum scabriculme* at both of the original collection sites, and we extend the number of known populations to nine for 16 total collections of the species. Nonetheless, *Trichophorum scabriculme* remains a very narrow endemic to the main range of the Hoàng Liên Mountains with just three collections known from outside Sa Pa District and two from outside Lào Cai Province.

Of the nine unique populations documented by specimens or pictures, only five are within protected areas (Hoàng Liên National Park), with several being either directly on the border with the park or near it (Ô Quy Hồ, Tiên Sa Waterfall). Even within the park, all of the sites are heavily influenced by human activity, as they are either primary tourist destinations (Tiên Sa and Silver Waterfalls, Phan Xi Păng) or they are in areas within the park where agriculture and grazing by farm animals is intense (site near Séo Mý Tảy village). Although *T. scabriculme* occurs in habitats that are typically difficult for animals and people to access (e.g., rock faces, steep streams), they are humid environments. It is thus vulnerable to any hydrological changes that result from the increased use of water for agriculture, fish farming or domestic use, which is common across its narrow range. At one of the Ô Quy Hồ sites where we collected *T. scabriculme* in 2012, significant changes in water flow have since occurred leading to algal buildup and the extirpation of at least one species of *Carex* collected there before (*C. hypolytroides*, Ford 1222 et al., WIN; Fig. 6). Three populations of *Trichophorum scabriculme* (Bình Lư, Ô Quy Hồ Pass and Văn Bàn) are immediately next to well-travelled roads through mountain passes (QL 4D and QL 279) that could be affected by road widening, especially for any increased traffic due to tourism. This is especially worrisome for populations along QL 4D and on mount Phan Xi Păng given the completion of a cable car to the summit in 2016. In addition, the authors know of only three populations where individuals were >50–100 individuals (Ô Quy Hồ Pass, site near Séo Mý Tảy village, type locality at Tiên Sa Waterfall) and only one herbarium label refers to the plants being “very common” (Than Uyên District) suggesting that the number of mature individuals is limited. We suspect that no more than 4,000 individuals currently exist.

Although we have significantly increased the number of known collections for the species and the size of its range, it still possesses an extent of occurrence of only 252.8 km^2^ over a north/south axis of just 47 km. Owing to its small extent of occurrence (<20,000 km^2^), limited number of populations (<10), and proximity to intense anthropogenic activity that could lead to the loss of populations or to a significant decline in the number of mature individuals in the near future, *Trichophorum scabriculme* is considered to be Vulnerable (VU) according to IUCN (2012) criterion B1 (a, b).

### Taxonomic treatment

#### Key to the species of *Trichophorum* in Vietnam and southern China (east of Tibet and south of the Chang Jiang or Yangzi River)

**Table:** 

**1a.** Culm fusiform to crescentiform in cross-section, with one sharp edge scabrous to base; bracts with long scabrous awns 4—10(—20) mm long; perianth bristles long exserted from glumes at maturity, forming a red cottony mass	***Trichophorum scabriculme***
**1b.** Culm terete to obtusely trigonous, smooth or scabrous only near apex; bracts with awns 0—5 mm long or absent; perianth bristles not much exserted from glumes or absent	**2**
**2a.** Inflorescence racemose to unispicate; perianth bristles present; all glumes in spikelets acute	***Trichophorum subcapitatum*agg.**
**2b.** Inflorescence strictly unispicate; perianth absent; proximal glume in spikelets mucronate, others obtuse	**3**
**3a.** Stigmas 3	***Trichophorum pumilum***
**3b.** Stigmas 2	***Trichophorum distigmaticum***

***Trichophorum scabriculme*** (Beetle) J. R. Starr, Lév.-Bourret & B. A. Ford ***comb. nov.*** Type: Indo-chine (Vietnam), Tonkin, Chapa. Parois siliceuses du ravin à la Cascade, vers 1,200 m. Février 1931, *A. Pételot* 6128 (holotype, GH!); isotypes P!, VNM (Photo!).

Basionym: *Scirpus scabriculmis* Beetle, *American Journal of Botany* 33: 665–666 (1946).

*Eriophorum scabriculme* (Beetle) Raymond (1957: 147).

*Description. —* Perennial herb, 10–60 cm tall, forming dense tufts of up to hundreds of culms. **Roots** smooth, faded yellow-brown to grey-brown, the central white strand surrounded by a thin ring of brown tissue and free from the rind. **Aerial vegetative parts** dark green. **Culms** pendant, crescentiform to fusiform in cross-section with a rounded edge and a sharp edge, 0.3–0.6 mm wide near base, antrorsely scabrous on the sharp edge with needle-like hyaline teeth 0.2—0.3 mm long, the sheath-clad base 1–1.6 mm wide including sheaths. **Leaves** all basal, but blades appearing cauline due to elongate sheaths. Distalmost leaf sheath 20–60 mm long, 0.7–1 mm wide at apex; inner bands very long, white-hyaline to brownish, apex somewhat inflated and indurated, white-hyaline with generally abundant red dots, at a 27–47° angle to the transverse plane of the culm; membranous ligule apparent as a projection of the leaf-sheath beyond leaf blade insertion, 0.2–0.4 mm long. Distalmost leaf blade 19–41 × 0.3–0.5 mm, involute-filiform, crescentiform in section, antrorsely scabrous with needle-like teeth on the margins from base to apex. **Inflorescence** unispicate or a very short raceme of 2–3 spikelets. Basal bracts sheathless, scale-like, with body 0.7–2 × 0.9–1.7 mm, with awn 4–10(–20) × 0.14–0.24 mm, flat, antrorsely scabrous on margins with needle-like barbs, rounded at the apex. Inflorescence always with 1–2 bracts at base. **Spikelets** narrowly elliptic when immature, 5–9 × 1.4–3 mm, becoming elliptic to obovate, the initially appressed glumes spreading at fructification; rachilla 0.4–0.7 mm wide including protruding receptacles, becoming dark-red with age, middle internodes 0.18–0.26 mm, receptacles upwardly open and protruding 0.1–0.2 mm from the rachilla axis; lateral spikelet prophyll empty, persistent, ovate to orbicular, ca. 1.3–2.2 × 0.9–1.5 mm, obtuse to bidentate at apex, completely encircling the pedicel at base but not sheathing, ca. 0.6–0.7 times as long as proximal glume, with 2 (rarely 3) major ribs reaching the teeth and several obscure red veins becoming indistinct below the middle, with several marginal barbules at the apex. **Glumes** ca. 14–18, deciduous, proximal glume sterile and sometimes awned, remainder all fertile and acute to shortly mucronate, proximal 2–3 smaller than the following, middle glumes triangular, 3–3.5 × 1.4–2 mm, 1.7–2 times as long as wide, mucronate to acute at apex, reddish-brown, chartaceous, margin undifferentiated; broad midrib pale, with a central prominent nerve reaching the apex; margins sometimes minutely barbed near the apex; glume wings extending around the flowers below their insertion point. **Flowers** bisexual, spiro-tristichously inserted; perianth bristles ca. 10–14, flat, pale tawny-brown to pale reddish-brown, sometimes with abundant red lines, to 25 mm long, much longer than nutlet, proximally smooth, but with minute antrorse barbs distally on margins and at apex, barbs hyaline and sharp; stamen 1, placed abaxially, the largest mature anthers ca. 1.5–1.6 mm long, with very short acute red apiculum, filament to ca. 1.5–3.8 mm long; style ca. 2.9–4 mm, red, 3–branched, the branches ca. 1.4–2.5 mm with abundant large papillae as long as wide. **Nutlets** 1.7–2.2 mm long, reddish-brown, surface minutely granular at maturity from silica-body projections; body fusiform, 1.5–1.9 × 0.4–0.7 mm, 2.5–4 times as long as wide, ca. 0.3–0.4 mm thick, plano-convex in section with a thickness/width ratio of ca. 0.5–0.7; beak clearly defined, 0.1–0.3 mm long including dark style remnant. **Embryo** narrowly turbinate in outline, with a basal root cap and lateral germ pore (Carex-type).

### Recognition

The type was originally identified as *Scirpus subcapitatus* Thwaites & Hook. (=*Trichophorum subcapitatum* (Thwaites & Hook.) D.A. Simpson), but it differs markedly from this species and all other southern Chinese and Vietnamese *Trichophorum* by culms scabrous on one edge from their base to apex versus culms smooth or scabrous apically only; by bracts with long scabrous awns (4–10 mm long) versus bracts lacking awns or shorter (0–5 mm long), and by fruits subtended by long bristles exsert from glumes versus bristles only slightly exceeding glume length or entirely lacking, amongst other characters. Like all *Trichophorum* species, it cannot be confused with *Eriophorum* or *Scirpus* because all its leaves are basal, and with highly reduced blades (except in *T. planifolium*), whereas species of *Eriophorum* and *Scirpus* have both well-developed basal and cauline leaves, with few exceptions.

*Trichophorum scabriculme* has also been provisionally determined as a *Carex* (*D.K. Harder et al. 6826*), a *Fimbristylis* (*A. Pételot 5498*, *Fimbristylis* cf. *pauciflora*; *Fimbristylis* sp. for all specimens in Hoàng Liên National Park Herbarium) and an *Eleocharis* (*N.T. Hiep, P.H. Hoang & L. Averyanov 2846*). This is understandable as it can superficially resemble single-spiked *Carex* species, and *Fimbristylis* and *Eleocharis* species with inflorescences composed of single spikelets. *Carex* can be distinguished from *T. scabriculme* by unisexual flowers and the presence of a perigynium; *Eleocharis* by distinct, thickened and persistent style-bases (tubercles) and eligulate leaves, and *Fimbristylis* by the absence of bristles and a deciduous style. It could also be possible to confuse *T. scabriculme* with *Isolepis* species that have just one or a few spikelets, but like *Fimbristylis* species, they lack bristles.

### Distribution

Việt Nam, Hoàng Liên Mountains, Lào Cai province from Sa Pa District south to Văn Bàn District and Lai Châu Province, from Tam Ðường District south to Tân Uyên District.

### Specimens examined

Because of the strong cultural influence of China, western travelers and trade, French colonialism, and the recent tumultuous period leading to full Vietnamese independence, place names and the political divisions of Vietnam have frequently changed. This means that multiple names and spellings typically exist for the same locality (McLeod & Nguyen, 2001; Woodside, 2006). For example, the modern tourist town of Sa Pa is spelt in most guidebooks as Sapa, whereas the French referred to it as Chapa or Cha-pa, and on the 1966 United States military map for Lào Kay (sheet 5753 I, series L7014; Lào Cai today), it is spelt Cha Pa. The French called the great pass west of Sa Pa that reaches 2,000 m Lö qui Hô (with many variants), but today it is formally known as Ô Quy Hồ and informally as Trạm Tôn, a name that refers to the fact the old ranger station had a metal roof over 20 years ago. Even the Vietnamese spelling of this common locality differs with the most frequent being Ô Quy Hồ whilst an administrative atlas of Vietnam (2008) writes it as Ô Quí Hồ. We know of at least seven different spellings for mount Phan Xi Păng, the highest mountain in Indochina. Moreover, because Vietnamese is a tonal language, but has a Latin alphabet, western herbarium labels almost always lack the diacritical marks that are the key to the pronunciation and meaning of words. In order to avoid future confusion, every effort was made to use the most common modern Vietnamese spelling with diacritical marks, even if this was not done on the label. Names in parentheses represent either common names used for the same locality (e.g., Tram Tôn or Ô Quy Hồ Pass), translations of the Vietnamese (e.g., Thác Bạc or “Silver Waterfall”), or a modern rendition of colonial names (e.g., Sa Pa for Chapa). Unless indicated, the latitude and longitudes given for specimens are accurate to <one km if not taken by GPS. The acronym “hlnph” stands for the Hoàng Liên National Park Herbarium, which contains approximately 8,000 well-curated specimens of the flora of the park (AT Vũ, pers. comm., 2016), but is not listed in Index Herbariorum.

VIỆT NAM. **Lào Cai Province**, Sa Pa District, Hoàng Liên National Park, Tram Tôn (Ô Quy Hồ Pass) (22°21′18.93″N, 103°46′27.25″E), 13 September 2005, *Trinh Dinh Hung* HL-165 (hlnph); Lào Cai Province, Sa Pa District, Hoàng Liên National Park, Tram Tôn (Ô Quy Hồ Pass) (22°21′18.93″N, 103°46′27.25″E), 18 July 2005, *Trinh Dinh Hung* 164 (hlnph); Lào Cai Province, Sa Pa District, Hoàng Liên National Park, Tram Tôn (Ô Quy Hồ Pass) (22°21′18.93″N, 103°46′27.25″E), 13 September 2005, *Trinh Dinh Hung* s.n. (hlnph); Lào Cai Province, Sa Pa District, Hoàng Liên National Park, Tram Tôn (Ô Quy Hồ Pass) (22°21′18.93″N, 103°46′27.25″E), 17 September 2005, *Trinh Dinh Hung* HL-161 (hlnph); Lào Cai Province, Sa Pa District, Hoàng Liên National Park, Sín Chảai (22°25′42.18″N, 103°47′54.85″E), *Tran Van Tu* TK050410–50, 4 April 2005 (hlnph). Lào Cai Province, Sa Pa District, Hoàng Liên National Park, Sín Chảai, *Vũ Anh Tái* TK050410–30, 4 April 2005 (hlnph). Lào Cai Province, Sa Pa District, Bảan Khoang (22°25′42.18″N, 103°47′54.85″E), *Dang Quyet Chien* s.n., 17 June 2005 (hlnph). Lào Cai, Văn Bàn District, Nạm Xé Commune (Municipality on label). Frequent, on vertical wall, at pass (22°02′26″N, 103°59′21″E), 612 m, 24 February 2001, *D.K. Harder et al.* 6826 (HN, MO); Lào Cai Province, Sa Pa District, Ô Quy Hồ Commune, Hoàng Liên National Park, on road to Lai Châu (QL 4D), ca. 10 km W of Sa Pa, ca. 100 m W of Thác Bạc (Silver Waterfall). Open rock face adjacent to waterfall (22°21′33.32″N, 103°46′39.48″, GPS), 1,831 m (GPS), 1,770 m (barometric altimeter), 10 April 2012, *B. Ford 1225, J. Starr and J. Regalado* (WIN). Lào Cai Province, Sa Pa District, Hoàng Liên National Park, Ô Quy Hồ Commune, on road to Lai Châu (QL 4D), ca. 10 km W of Sa Pa, ca. 300 m W of Thác Bạc (Silver Waterfall). Rock fissures and faces with seepage, adjacent to and at bottom of waterfall, plants growing from a moss substrate (22°21′19.2″N, 103°46′25.8″E, GPS [WGS 84]), 1,850 m (barometric altimeter), 10 April 2012, *B. Ford 1227, J. Starr and J. Regalado* (WIN). Lào Cai Province, Sa Pa District, Tảa Van Commune, Hoàng Liên National Park, gravel road to Séo Mý Tảy village. Growing in moss pockets on narrow rock ledges and fissures along fast moving stream (22°15′37.9″N, 103°53′24.2″E, GPS [WGS 84]), 1,716 m (GPS), 15 April 2012, *B. Ford 1256, J. Starr, J. Regalado, Vũ Anh Tái, and Nguyê˜n Kim Thanh* (WIN). Lào Cai Province, Sa Pa District, Hoàng Liên National Park, Tram Tôn Trail (main trail) to summit of Mount Phan Xi Păng (Fansipan) between “2900 Pass” and summit, open, NE facing moist granitic rock face with moss covered ledges and pockets (22°18′26.9″N, 103°46′17.9″E, GPS [WGS 84]), 2,878 m (GPS), 2,760 m (altimeter), 21 April 2015 , *B. Ford* 15080, *J. Starr, É. Léveillé-Bourret, Nguyê˜n Thị Kim Thanh, Vũ Anh Tài, and S. Ford* (WIN). Lào Cai Province, Sa Pa District, Hoàng Liên National Park, Cát Cát, trail along suôi Vàng (Gold Stream), in the vicinity of Cát Cát Waterfall (Thác Tiên Sa or “La Cascade” in French), right bank of suô´i Vàng in fissures of moist granitic rock faces above and along stream (22°19′18.8″N, 103°49′34.4″E, GPS [WGS 84]), elevation 1,245 m (GPS), 1,150 m (altimeter), 24 April 2015, *B. Ford* 15089*, J. Starr, É. Léveillé-Bourret, Nguyê˜n Thị Kim Thanh, Vũ Anh Tài, and S. Ford* (WIN); Tonkin (Northern Vietnam): Chapa (Sa Pa, Lào Cai Province). Parois siliceuses du ravin à la Cascade (22°19′38.57″N, 103°50′04.28″N, visited), vers 1,200 m. février 1931, *A. Pételot* 6128 (P, GH; VNM, photo!); Tonkin: Chapa, Col de Lö qui Hô (Ô Quy Hồ Pass), fissures des rochers calcaires très ensoleillés, vers 2,200 mètres (22°21′12.28″N, 103°46′26.64″E), avril 1944, *A. Pételot* 8635 (MT, L); Tonkin: Chapa. Sur rochers siliceux au milieu d’un torrent près du gué Ysault, vers 1,300 m, environs de Chapa, février 1931, *Pételot* 5498 (P) (locality unknown). **Lai Châu Province**, Tam Ðường District, Bình Lư Commune, on road to Bình Lư (QL 4D), ca. 11.4 km W of Sa Pa by air or 22 km by road, SE facing weeping granitic rock face along highway, soil pH 6.7 (22°21′31.2″N, 103°44′02.5″E, GPS[WGS 84]), 1,546 m (GPS), 18 April 2015, *B. Ford 15053, J. Starr, É. Léveillé-Bourret, Nguyê˜n Thị Kim Thanh, Vũ Anh Tài, and S. Ford* (WIN); Lai Châu Province (Lào Cai Province on label), Than Uyên District (now Tân Uyên District), Hồ Mít Commune (Municipality on label). Lithophytic sedge on open wet vertical bluffs—very common (22°06′N 103°52′E; mapped as 22°6′49.5 N, 103°55′33.4″E based on label description, but >one km inaccuracy), 1,700–2,200 m, 21 May 1999, *N.T. Hiep, P.H. Hoang & L. Averyanov* 2846 (MO).

### Habitat

Mountains (612 m to 2,878 m) in full sun on vertical rock walls and road cuts, along steep cascading streams and on the edges and foot of waterfalls where moisture is present. Plants typically grow within a moss substrate. *Carex hypolytroides* and *Scirpus ternatanus* Reinwardt ex Miquel were seen at all localities near Sa Pa where this species was collected (*Ford et al. 1225, 1227, 1256*).

### Conservation status

Vulnerable (VU) category of IUCN (2012) based on criterion B1 (a, b). Only nine populations are known (<10) and the extent of occurrence is 252.8 km^2^ (<20,000 km^2^). Owing to intense anthropogenic activity near most populations (roads, agriculture, fish farming, tourism), there is reason to believe that subpopulations could be lost or a significant decline in the number of mature individuals could occur in the near future.

### Etymology

The epithet *scabriculme* combines the Latin word for rough or scabrous (*scabri*-) with the Latin for culm (*culmus*) in reference to the culms of *T. scabriculme* that are scabrous from their base to their apex, a character that is unique in *Trichophorum*.

### Notes

In addition to the localities noted above, plants were also seen at two additional localities in the Sa Pa District of Lào Cai Province. As these plants were either too mature or too immature to make adequate herbarium specimens, they were not collected, but georeferenced photographs were taken to document their existence. In the interests of conservation, these localities are briefly described as follows (localities are very close to collections cited above): (1) Immature plant on steep edge of Thác Bạc (Silver Waterfall) (22°21′44.69″N, 103°46′38.15″E), 10 April 2012, 1,733 m (GPS); (2) large population of overly mature plants along south facing rock wall of the road (QL 4D) to Ô Quy Hồ Pass (22°21′21.67″N, 103°46′33.07″E), 10 April 2012, 1,882 m (GPS). Note that these two localities demonstrate how the maturity of specimens can vary widely over short distances if there are significant differences in hydrology and sun exposure.

*Beetle’s (1946)* translation of the French label data on the holotype (Pételot 6128) “Indo-China, Chape, rocks of ravine, Feb. 1931” is incomplete and does not help with determining the type locality. The label is translated here as “Indochina, Tonkin, Chapa. Siliceous walls of the ravine at the Cascade, around 1,200 m, February 1931”. During the French Colonial period, “La Cascade” was a popular tourist attraction near Sa Pa (Chapa) that was described, mapped and pictured in tourist guides (Anonymous, 1924; Anonymous, 1932). “La Cascade” is here identified as Tiên Sa Waterfall (N22°19′38.57″, E103°50′04.28″) near Cát Cát village, which is approximately two km by road from Sa Pa and continues to be a popular tourist attraction today. “La Cascade” can be definitively identified as the type locality thanks to the presence of a hydroelectric power station constructed by the French in 1925 that is mentioned in one guide (Anonymous, 1932) and still exists at the site to this day. Moreover, the elevation of the site (ca. 1,237 m) is consistent with the holotype’s label (around 1,200 m). When we visited the site in 2015, *Trichophorum scabriculme* could still be seen growing on the rock faces opposite the main terrace for tourists to view the waterfalls. From this position, a population consisting of thousands of plants could be seen on a rock face approximately 200 m upstream and 30 m above the right bank of the suô´ Vàng. This is the largest population known to us. The name of the modern village of Cát Cát has no meaning in Vietnamese and is mostly likely derived from the French word “Cascade” for the waterfalls at this locality.

## Conclusions

Although *Trichophorum scabriculme* possesses a unique combination of characters within sedges, DNA data, morphology and embryology strongly support its position within the Scirpo-Caricoid Clade including its placement within the genus *Trichophorum*. *Trichophorum* now consists of 14 species, but it is likely that future studies will conclude that its limits include species of *Cypringlea* and *Oreobolopsis* as well. *Eriophorum* s.str. now consists of approximately 18 species, but it is likely that future studies will find that the aberrant *E. transiens* is best aligned with elements in the Ficinia Clade. *Eriophorum* is nested in *Scirpus* s.str. but further research is required to determine whether the limits of *Scirpus* are appropriately defined.

Despite discovering seven new populations and extending its range westward to Lai Châu Province and southward in Lào Cai Province by more than 47 km, the conservation status of *Trichophorum scabriculme* in Vietnam is Vulnerable (VU). Only 56% of populations are found in protected areas and intense anthropogenic activity continues to threaten the existence of this unique sedge species.

##  Supplemental Information

10.7717/peerj.7538/supp-1 Table S1 Vouchers and Genbank accession numbers for samples used in the molecular phylogenetic analysis of the Scirpo-Caricoid Clade and *Trichophorum scabriculme* Taxa are in alphabetical order with outgroups last.

10.7717/peerj.7538/supp-2 Data S1 Sequence data (*matK* and *ndhF*) used in phylogenetic analyses of *Eriophorum scabriculme*

10.7717/peerj.7538/supp-3 Data S2 Geolocated locality data for all known *Trichophorum scabriculme* for use with the Geospatial Conservation Assessment Tool (GeoCAT)

## Additional Information and Declarations

### Field Study Permissions

The following information was supplied relating to field study approvals (i.e., approving body and any reference numbers): Field collection permits were issued by the Vietnamese Forest Protection Department under the support of the faculty and staff at VNU University of Science, Hanoi (Permits numbers 222/TCLN-BTTN (2012) and 184/TCLN-BTTN (2015)).

### DNA Deposition

The following information was supplied regarding the deposition of DNA sequences: The DNA matrix is available as a Supplemental File.

### Data Availability

The following information was supplied regarding data availability: The raw data is available at GenBank (Table 1) and as a data matrix for analysis in the Supplemental File.

### Publication LSID

*Trichophorum scabriculme* (Beetle) J.R.Starr, Lév.-Bourret&B.A. Ford LSID 77200603-1.
