# Supplementary material for: The rediscovery of the rare Vietnamese endemic Eriophorum scabriculme redefines generic limits in the Scirpo-Caricoid Clade (Cyperaceae)
Source: PeerJ. 2019 Sep 25;7:e7538. doi: 10.7717/peerj.7538 (PMC6765354; doi:10.7717/peerj.7538)
Supplement: Table S1 — Taxa are in alphabetical order with outgroups last. [file peerj-07-7538-s001.docx]

| **Species** | **DNA number** | **Collectors** | **Coll. number** | **Herb.** | **Origin** | ***matK*** | ***ndhF*** |
| --- | --- | --- | --- | --- | --- | --- | --- |
| *Amphiscirpus nevadensis* (S.Watson) Oteng-Yeb. | STA2141 | Hudson | 5177 | CAN | Canada | JX065075 | JX074631 |
| *Blysmus compressus* (L.) Panz. ex Link | STA1902 | Kotowicz | 871 | CAN | Poland | KJ513577 | KJ513482 |
| *Blysmopsis rufa* (Huds.) Oteng-Yeb. | STA1913 | Jokela | 9-VIII-1958 | CAN | Finland | JX065076 | JX074632 |
| *Calliscirpus brachythrix* C.N.Gilmour et al. | STA2132 | Janeway | 6344 | CHSC | United States | JX074667 | KJ513486 |
| *Calliscirpus criniger* (A. Gray) C.N.Gilmour et al. | STA1898 | Tracy | 9380 | DAO | United States | JX074654 | KJ513487 |
| *Carex acicularis* Boott | STA__84 | Ford | 29/94 | CHR | New Zealand | KJ513581 | KJ513489 |
| *Carex aphylla* Kunth | STA1279 | Starr & Villaverde | 10025 | CAN | Argentina | KJ513582 | KJ513490 |
| *Carex blanda* Dewey | STA_183 | Bakowsky | 96-176 | WIN | Canada | KJ513583 | KJ513491 |
| *Carex ecuadorensis* (G.A.Wheeler & Goetgh.) J.R.Starr | STA_148 | Starr & Amigo | 99020 | FHO | Ecuador | KJ513667 | KJ513574 |
| *Carex gynocrates* Wormsk. ex Drejer | STA_817 | Ford & al. | 02283 | WIN | Canada | KJ513587 | KJ513495 |
| *Carex lancea* Dewey | STA_710 | Dahlstrand & McDonald | 1302 | PRE | South Africa | KJ513625 | KJ513532 |
| *Carex polystachya* Sw. ex Wahlenb. | STA_210 | Jones & Wipff | 11275 | MICH | Belize | KJ513589 | KJ513497 |
| *Carex pulicaris* L. | STA_105 | Starr & Scott | 98001 | FHO | England | KJ513590 | KJ513576 |
| *Carex siderosticta* Hance | STA_733 | Léveillé-Bourret | 545 | CAN | Garden | KJ513592 | KJ513499 |
| *Carex simpliciuscula* Wahlenb. | STA1801 | Porsild | 1825 | CAN | Canada | JX065088 | JX074644 |
| *Carex stipata* Muhl. ex Willd. | STA1808 | Dugal & Camfield | 3728 | CAN | Canada | KJ513593 | KJ513500 |
| *Cypringlea analecta* (Beetle) M.T.Strong | STA2052 | Reznicek & al. | 11094 | MICH | Mexico | KJ513594 | KJ513501 |
| *Cypringlea evadens* (C.D.Adams) Reznicek & S.González | STA2053 | Rawlins & Sholes | 2830 | MICH | Mexico | JX065082 | JX074638 |
| *Dulichium arundinaceum* (L.) Britton | STA_154 | Ford & Punter | 94233 | FHO | Canada | JX065083 | JX074639 |
| *Eriophorum angustifolium* Honck. subsp. *angustifolium* | STA1777 | Scoggan | 10947 | CAN | Canada | KJ513597 | KJ513504 |
| *Eriophorum callitrix* Cham. | STA1786 | Porsild & Porsild | 4753 | CAN | Canada | JX074653 | JX074641 |
| *Eriophorum gracile* W.D.J.Koch ex Roth | STA_393 | Starr & Thibeault | 6014 | CAN | United States | KJ513605 | KJ513512 |
| *Eriophorum latifolium* Hoppe | STA2051 | Jokela & Paavo | 20-VII-1965 | OSC | Finland | KJ513606 | KJ513513 |
| *Eriophorum russeolum* Fr. subsp. *russeolum* | STA1793 | Gauthier | 75-208 | CAN | Canada | KJ513608 | KJ513515 |
| *Eriophorum scheuchzeri* Hoppe subsp. *scheuchzeri* | STA1798 | Argus & Chunys | 5813 | CAN | United States | KJ513611 | KJ513518 |
| *Eriophorum tenellum* Nutt. | STA1928 | Dugal & Shchepanek | 6354 | CAN | Canada | KJ513612 | KJ513519 |
| *Eriophorum vaginatum* L. subsp. *vaginatum* | STA_112 | Starr & Scott | 98007 | FHO | England | KJ513615 | KJ513522 |
| *Eriophorum virginicum* L. | STA1807 | Dickson & Brunton | 3214 | CAN | Canada | KJ513617 | KJ513524 |
| *Eriophorum viridicarinatum* (Engelm.) Fernald | STA1780 | Shea | 11351 | CAN | Canada | JX074652 | JX074640 |
| *Khaosokia caricoides* D.A.Simpson et al. | STA_387 | Middleton & al. | 4071 | MICH | Thailand | JX065087 | JX074643 |
| *Oreobolopsis tepalifera* T.Koyama & Guagl. | STA2038 | Wood | 10463 | NY | Bolivia | JX065089 | JX074645 |
| *Phylloscirpus deserticola* (Phil.) Dhooge & Goetgh. | STA2101 | Solomon | 15819 | CAS | Bolivia | KJ541072 | KJ541073 |
| *Rhodoscirpus asper* (J.Presl & C.Presl) Lév.-Bourret et al. | STA2173 | Kiesling | 10341 | SI | Argentina | KP165402 | KP212422 |
| *Scirpus cyperinus* (L.) Kunth | STA1773 | Lindsay | 1025 | CAN | Canada | JX065092 | JX074648 |
| *Scirpus divaricatus* Elliott | dS014 | Spalink | 124 | WIS | United States | KJ513633 | KJ513540 |
| *Scirpus hattorianus* Makino | STA1984 | Bergeron & al. | 81-111 | CAN | Canada | KJ513640 | KJ513547 |
| *Scirpus karuisawensis* Makino | dS004 | Jung | 807017 | AJOU | South Korea | KJ513641 | KJ513548 |
| *Scirpus longii* Fernald | dS052 | Spalink | 251 | WIS | United States | KJ513642 | KJ513549 |
| *Scirpus maximowiczii* C.B.Clarke | STA1920 | Petrochenko | 357 | CAN | Russia | KJ513644 | KJ513551 |
| *Scirpus microcarpus* J.Presl & C.Presl | STA1976 | Dugal, & Camfield | 3770 | CAN | Canada | KJ513646 | KJ513553 |
| *Scirpus pallidus* (Britton) Fernald | STA1983 | Hudson | 5079 | CAN |  | KJ513647 | KJ513554 |
| *Scirpus pendulus* Muhl. | STA1978 | Cruise | 1388 | CAN | Canada | KJ513649 | KJ513556 |
| *Scirpus polyphyllus* Vahl | dS050 | Spalink | 246 | WIS | United States | KJ513650 | KJ513557 |
| *Scirpus polystachyus* F.Muell. | STA_349 | Pullen | 4091 | A | Australia | KJ513651 | KJ513558 |
| *Scirpus radicans* Schkuhr | STA1915 | Samuelsson | 296 | CAN | Sweden | KJ513653 | KJ513560 |
| *Scirpus sylvaticus* L. | dS005 | Jung | 806038 | AJOU | South Korea | KJ513654 | KJ513561 |
| *Scirpus wichurae* Boeckeler | dS003 | Jung | 808322 | AJOU | South Korea | KJ513655 | KJ513562 |
| *Sumatroscirpus rupestris* Lév.-Bourret & J.R.Starr | STA2769 | Ford & al. | 15081 | WIN | Vietnam | MF669228 | MF669289 |
| *Trichophorum alpinum* | STA1815 | Spetzman | 4941 | CAN | United States | JX065093 | JX074649 |
| *Trichophorum alpinum* (L.) Pers. | STA2047 | Cayouette & al. | 75-78 | CAN | Canada | KJ513656 | KJ513563 |
| *Trichophorum cespitosum* (L.) Hartm. | STA1819 | Aiken & Iles | 02-048 | CAN | Canada | KJ513657 | KJ513564 |
| *Trichophorum cespitosum* (L.) Hartm. | STA1817 | Saarela & Percy | 1219 | CAN | Canada | JX065094 | JX074650 |
| *Trichophorum clintonii* (A.Gray) S.G.Sm. | STA_1822 | Pratt, P D | 128 | CAN | Canada | KJ513658 | KJ513565 |
| *Trichophorum pumilum* (Vahl) Schinz & Thell. | STA1820 | Bennett & al. | 06-097 | CAN | Canada | KJ513659 | KJ513566 |
| *Trichophorum pumilum* (Vahl) Schinz & Thell. | STA1922 | Mejland | 5-VII-1963 | CAN | Norway | KJ513660 | KJ513567 |
| *Trichophorum rigidum* (Boeckeler) Goetgh. et al. *subsp. rigidum* | STA2043 | Unknown | 1102 | NY | Bolivia | KJ513661 | KJ513568 |
| *Trichophorum rigidum* (Boeckeler) Goetgh. et al. *subsp. rigidum* | STA2078 | Ritter & Wood | 2832 | A | Bolivia | KJ513662 | KJ513569 |
| *Trichophorum scabriculme* (Beetle) J.R.Starr et al. | STA2401 | Ford & al. | 1225A | WIN | Vietnam | **KX588070** | **KX588075** |
| *Trichophorum scabriculme* (Beetle) J.R.Starr et al. | STA2400 | Ford & al. | 1225B | WIN | Vietnam | **KX588071** | **KX588076** |
| *Trichophorum scabriculme* (Beetle) J.R.Starr et al. | STA2408 | Ford & al. | 1227A | WIN | Vietnam | **KX588072** | - |
| *Trichophorum scabriculme* (Beetle) J.R.Starr et al. | STA2451 | Ford & al. | 1256A | WIN | Vietnam | **KX588073** | **KX588077** |
| *Trichophorum subcapitatum* (Thwaites & Hook.) D.A.Simpson | STA2102 | Tucker | 15100 | US | China | **KX588069** | **KX588074** |
| *Trichophorum subcapitatum* (Thwaites & Hook.) D.A.Simpson | STA2070 | Luo | 1903 | CAS | China | KJ513663 | KJ513570 |
| *Trichophorum uniflorum* (Trautv.) Malyschev & Lukitsch. | STA1917 | Malishev | 27-VII-1950 | CAN | Russia | KJ513664 | KJ513571 |
| *Trichophorum uniflorum* (Trautv.) Malyschev & Lukitsch. | STA1918 | Ivanova & Moskvin | 756 | CAN | Russia | KJ513665 | KJ513572 |
| *Zameioscirpus atacamensis* (Phil.) Dhooge & Goetgh. | STA2143 | Ru | 9884 | US | Argentina | JX065095 | JX074651 |
| *Abildgaardia ovata* (Burm.f.) Kral | STA_328 | Muasya & al. | 684 | K | Kenya | JX065086 | JX074642 |
| *Bulbostylis atrosanguinea* (Boeckeler) C.B.Clarke | STA_321 | Muasya | 1037 | K | Kenya | KJ513580 | KJ513485 |
| *Eleocharis acicularis* (L.) Roem. & Schult. | dS484 | Fields | 2583 | WIS | United States | KJ513595 | KJ513502 |
| *Erioscirpus comosus* (Wall.) Palla | STA2092 | Hing & al. | 22413 | A | China | KJ513619 | KJ513526 |
| *Isolepis aucklandica* Hook.f. | STA1906 | McIntosh | 12-II-1977 | CAN | New Zealand | KJ513621 | KJ513528 |
